# The effect of radiotherapy on fat engraftment for complete breast reconstruction using lipofilling only

**DOI:** 10.1007/s00404-022-06610-4

**Published:** 2022-05-30

**Authors:** Norbert Heine, Andreas Eigenberger, Vanessa Brebant, Sally Kempa, Stephan Seitz, Lukas Prantl, Britta Kuehlmann

**Affiliations:** 1 Aesthetic, Hand and Reconstructive Surgery, University Center for Plastic, University Hospital Regensburg, Franz-Josef-Strauß-Allee 11, 93053 Regensburg, Germany; 2 Faculty of Mechanical Engineering, Ostbayerische Technische Hochschule Regensburg (OTH Regensburg), Regensburg, Germany; 3 Department of Obstetrics and Gynecology, Caritas Hospital St. Josef, University of Regensburg, Regensburg, Germany

**Keywords:** Breast augmentation, Radiotherapy, Breast cancer, Lipofilling, Fat graft, Reconstruction

## Abstract

**Purpose:**

Lipofilling has been established as a standard technique for contour enhancement following breast reconstruction. However, there is a paucity in current literature regarding the use of this technique for complete reconstruction of the female breast as an alternative to conventional techniques, such as expander or flap-based procedures. In particular, the influence of pre-operative irradiation for successful reconstruction has rarely been examined in published studies. Here, the authors describe their experience with successful fat injection in pre-radiated breasts in comparison with non-pre-radiated patients.

**Methods:**

In this retrospective study, we examined a total of 95 lipofilling treatments on 26 patients (28 breasts). All of them experienced mastectomy following breast cancer; local breast defects after partial resection of the gland were not included in this study. In total, 47 lipofilling procedures in 12 non-irradiated patients (14 breasts) and 48 procedures in 14 irradiated women (also 14 breasts) were performed. Per session, approximately 297 ± 112 cc of adipose tissue was grafted in group A (no radiotherapy) and approximately 259 ± 93 cc was grafted in group B (radiotherapy).

**Results:**

Among the group of women without pre-operative radiation, 71% of breast reconstructions limited to lipofilling only showed constant engraftment of fat tissue with a successful reconstructive result, whereas only 21% of the patients with pre-radiated breasts showed complete reconstruction of the breast with a permanent fat in-growth.

**Conclusion:**

Preoperative radiotherapy significantly impedes successful completion of breast reconstructions planned only by autologous fat transfer. Patients should be selected individually and carefully for complete breast reconstruction using lipofilling only.

## Introduction

In addition to the gold standards of free and pedicled flaps and expander- or implant-based techniques, reconstruction of the female breast by lipografting has been established during the last decade as an alternative method for selected indications [1–3]. Using this method, fat harvested by liposuction is injected to improve contour deformities after breast conserving procedures or, in highly selected cases, for complete reconstruction after breast surgery.

While fat transplantation into the breast was considered to be at higher risk for radiologic detection and breast cancer recurrence in the past (ASPS ad hoc committee 1987), Petit et al. [4] published a multi-center study in 2011 including 513 patients demonstrating oncologic safety of fat grafting for breast reconstruction. A detailed workup of this study in 2013 recognized an increased risk for patients after ductal carcinoma in situ (DCIS) [5] but 2016 Kronowitz et al. [6] confirmed the overall safety of this method in a matched controlled study with 719 women.

The effects of pre-operative irradiation on soft tissue in breast reconstruction are undeniable [7]. Furthermore, some studies have reported over 50% of major complications after expander- or implant-based reconstructions in combination with radiotherapy [8].

In contrast, reconstruction with autologous tissue using flap surgery (e.g., DIEP flap) is less often subject to negative sequelae following irradiation [9], with volume loss from irradiation also expected in flaps with adequate blood supply [10]. While the consequences of pre-operative and postoperative radiotherapy for conventional techniques of breast reconstruction are well investigated, only few studies are published regarding the effect of irradiation on partial or complete breast reconstruction by lipofilling [11, 12].

Several papers have already described the use of lipofilling to improve contour deformities in irradiated patients, hereby pointing out the advantage of lipofilling in improving tissue quality [13, 14]. Rigotti et al. described that purified autologous lipoaspirates (60–120 cc), including isolated stromal vascular fraction and thus cells with mesenchymal stem cell physical properties and immunophenotype help to resolve the late side effects of radiotherapy by enhancing progressive regeneration [15]. The pre-treatment of irradiated skin with fat grafting prior to implant-based reconstruction was evaluated positively as well [16].

The aim of the present study was to compare the results of lipografting for complete breast reconstruction after mastectomy in a retrospective study in both irradiated and non-irradiated patients.

## Materials and methods

Between May 2009 and May 2014, 26 mastectomy patients (28 breasts) were transferred to our department for complete reconstruction by lipofilling only. Exclusion criteria were a BMI lower than 22, active tumor disease, ongoing or planned chemotherapy, and genetic disorder (BRCA 1/2). All patients were informed of the advantages and disadvantages of other possible breast reconstruction procedures with their advantages and disadvantages. Indication for fat grafting as sole procedure performed after mastectomy was based upon the patient’s request after informed consent. Intended outcome of these treatments was complete reconstruction of the ablated breast using lipofilling only without further reconstructive techniques, such as flap surgery or implants. The desired breast size was defined by the size of the opposite breast side or, in case of absolute or relative macromastia, by the patient's desired breast size.

Fat grafting was performed using a 3 mm, blunt-edge cannula and harvested using Tissu-Trans Filtron^®^ (Shippert Medical Technologies Corporation, Colorado). Using a small-gage cannula of 2 mm to reduce trauma to the recipient site, processed fat was reinjected during the same surgery. Approval was given by the local ethic committee of university clinic in Regensburg, vote no. 18-1226-101, following the ethical standards of the Helsinki Declaration of 1975, as revised in 2000.

In this retrospective study, a total of 95 fat graftings were performed. Health Insurance covered all procedures. Patients were divided into two groups: Group A without a history of radiotherapy and Group B with pre-operative irradiation.

In Group A without radiotherapy, 12 patients (with 14 breasts after mastectomy) were enrolled for a total of 47 lipofilling procedures. On average, 3.4 individual surgeries were performed per breast; a minimum of one lipofilling and a maximum of eight lipofillings per patient were carried out. Two women received lipofilling as treatment for both their breasts. One of these two patients required eight separate lipofilling sessions per side until successful reconstruction, whereas the other one needed two procedures to successfully reconstruct the volume of both breasts. In general, the right breast side was treated 32 times while the left side was treated 15 times among this group. On average, 297 ± 112 cc of fat per breast per surgery was injected (min. 40 cc, max. 500 cc). Patients were between 44 and 70 years old (median 52 years) at the time of the first surgery. The average body weight was 68.6 ± 12.0 kg (range 53–89 kg) and the average height was 165 ± 6 cm (range 157–173 cm). The average BMI was 25.1 ± 3.0. Nine out of the 12 women had used the BRAVA system as support (75%; Table 1). This BRAVA-device (which is no longer available on the market) works as a negative-pressure external expander to enlarge the potential recipient tissue for the injected fat, and has to be worn for 4–6 weeks before the operation.

**Table 1:** Descriptive statistics (Group A and Group B)

	Group A		Group B	
	Mean (± SD)	Range	Mean (± SD)	Range
Age^a^ [years]	54 (± 8.2)	44–70	52 (± 6.5)	42–66
BMI^a^ [kg/m^2^]	25.1 (± 3)	21–31	24.3 (± 3.4)	19–29
Height [cm]	165 (± 6)	157–173	165 (± 67)	155–176
Weight^a^ [kg]	68.6 (± 12)	53–89	66.5 (± 19.5)	54–86
Number of operations	3.4 (± 1.9)	1–8	3.4 (± 2.3)	1–9
Injection volume^b^ [ml]	297 (± 112)	40–500	259 (± 93)	60–500
Chemotherapy	4		10	
Minor complications (oil cysts)	2		1	

^a^At the time of first breast surgery

^b^Per treatment

In Group B (pre-operative radiation in their medical history), 14 patients were treated for a total of 48 interventions. On average, one to nine separate procedures (mean 3.4 ± 2.3 procedures) were performed per patient, with 27 operations on the right breast side and 21 operations on the left. No patient was treated on both breast sides. On average, 259 ± 93 cc of adipose tissue was injected (range 60–500 cc). Women were between 42 and 66 years old (median 53 years) at the time of the first surgery. Average weight was 66.5 ± 19.5 kg (range 54–86 kg); size was 165 ± 6 cm (range 158–176 cm), the average BMI was 24.3 ± 3.4. The BRAVA system was worn by 11 patients (79%) (Table 1).

Statistical analysis was mainly descriptive. Since our data in terms of completed breast reconstruction are binary (successful, unsuccessful), we applied the Fisher’s exact test for the assessment of significant difference between the two groups (with and without pre-radiation) using R Studio Team (PBC, Massachusetts). *P* values < 0.05 were considered as statistically significant.

## Results

For this study, 26 post-mastectomy women were enrolled who were scheduled for complete breast reconstruction limited to autologous lipofilling. 12 patients with in total 14 breasts were treated and had no history of radiotherapy, 14 women were treated with pre-irradiation of the chest wall. Women in both groups were comparable in height, weight, BMI, and age, which are shown in Table 1. On average, more volume was infiltrated per operation in Group A (no radiotherapy) than in Group B (radiotherapy) (297 vs. 259 cc) (Table 1).

**Fig. 1:**
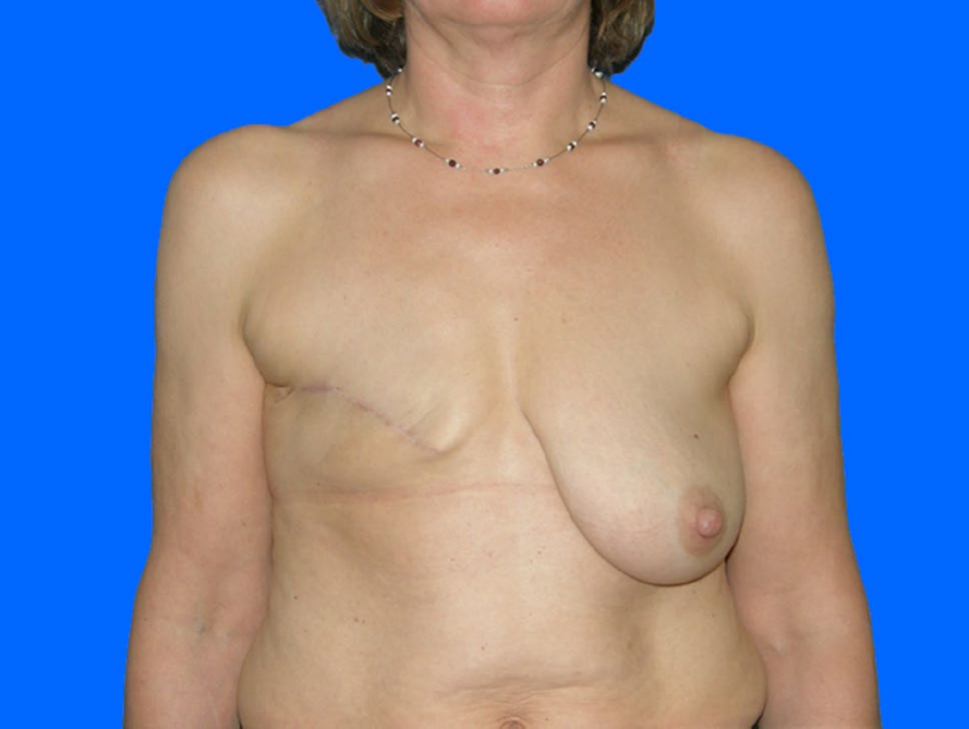
54 years old patient of Group A (without radiotherapy), pre-operative

**Fig. 2:**
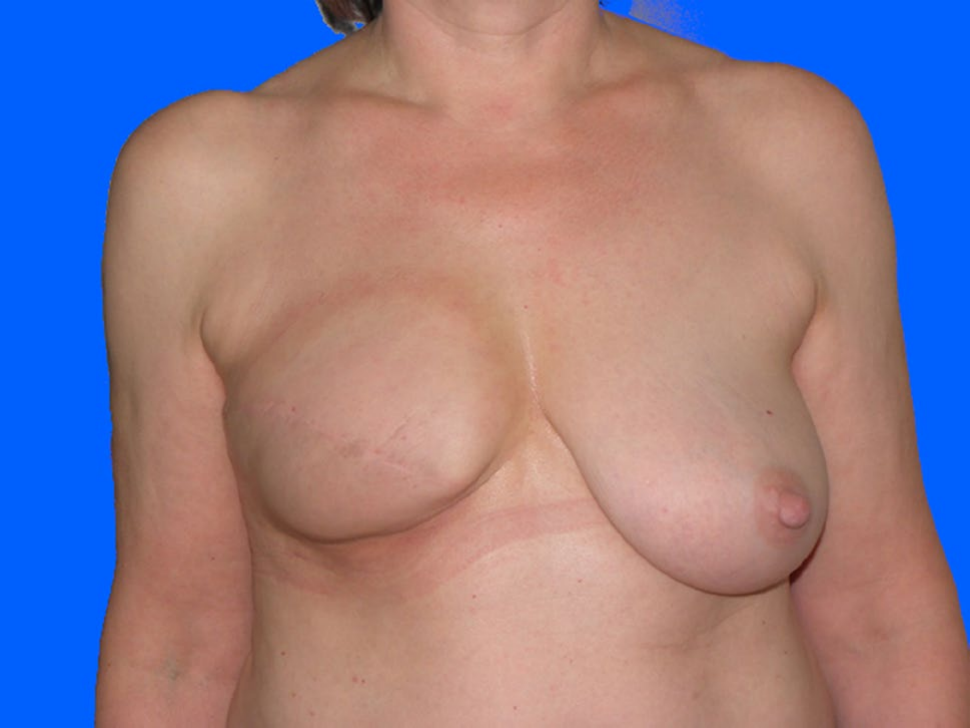
Same patient of Group A (without radiotherapy), postoperative after three sessions lipofilling (380, 285 and 380 ml injection volume per session)

**Fig. 3:**
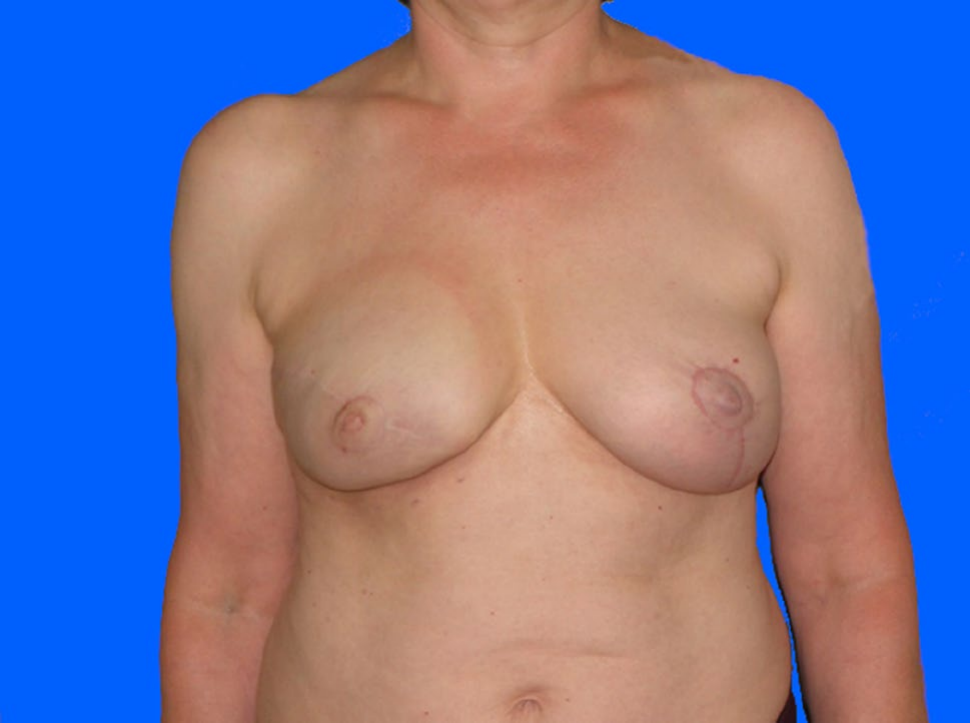
Same patient of Group A (without radiotherapy), postoperative after NAC reconstruction and mastopexy on contralateral breast

Within the 14 breasts in Group A (12 patients; two after bilateral mastectomy), complete breast reconstruction was achieved in nine women (ten breasts, 71%) (Figs. 1, 2, 3). Here, a mean of 3.4 individual surgeries was required for ultimate success (min. two, max. eight).

In two cases, a change of procedure to expander or implant was performed. In one case of bilateral mastectomy, the treatment was finalized early without finishing the preferred outcome and no further reconstruction was performed.

Four patients (five breasts) received chemotherapy before lipofilling; none of the women had a history of smoking or diabetes.

As minor complications, oil cysts were reported in two cases.

In Group B, including 14 women who had undergone pre-operative radiotherapy, complete reconstruction was achieved in three cases (three breasts, 21%), requiring three (one patient) or four sessions (two patients) for complete reconstructive success.

In average, the time interval between radiotherapy and the first lipofilling session was 7 years (range 1–16 years).

**Fig. 4:**
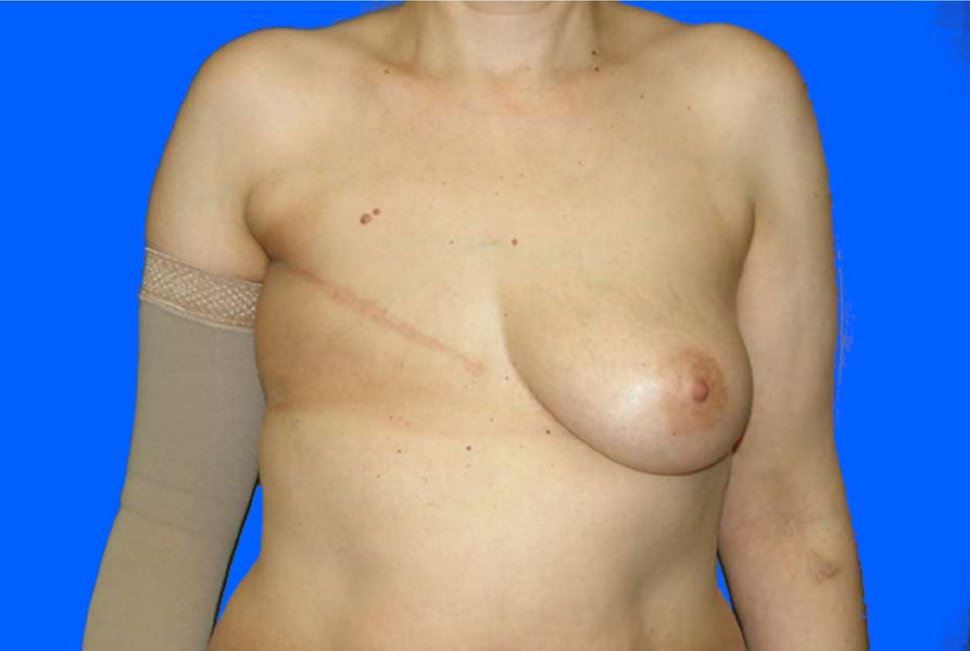
46 years old patient of Group B (with radiotherapy), pre-operative

**Fig. 5:**
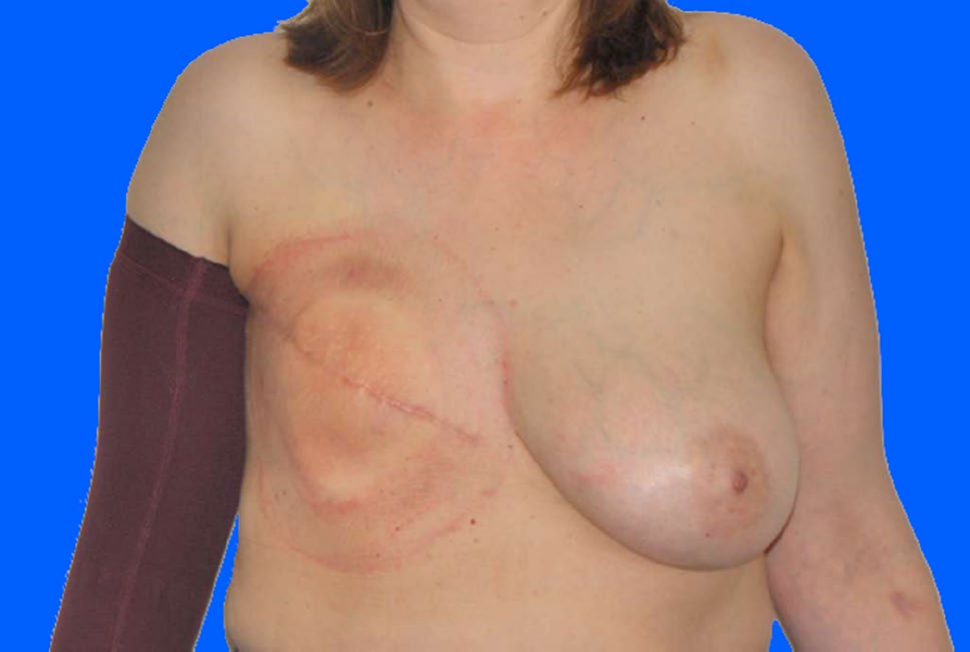
Same patient of Group B (with radiotherapy), postoperative after four sessions lipofilling (320, 320, 200 and 300 ml injection volume per session) and BRAVA treatment (indicated by the red circle around the left thorax)

**Fig. 6:**
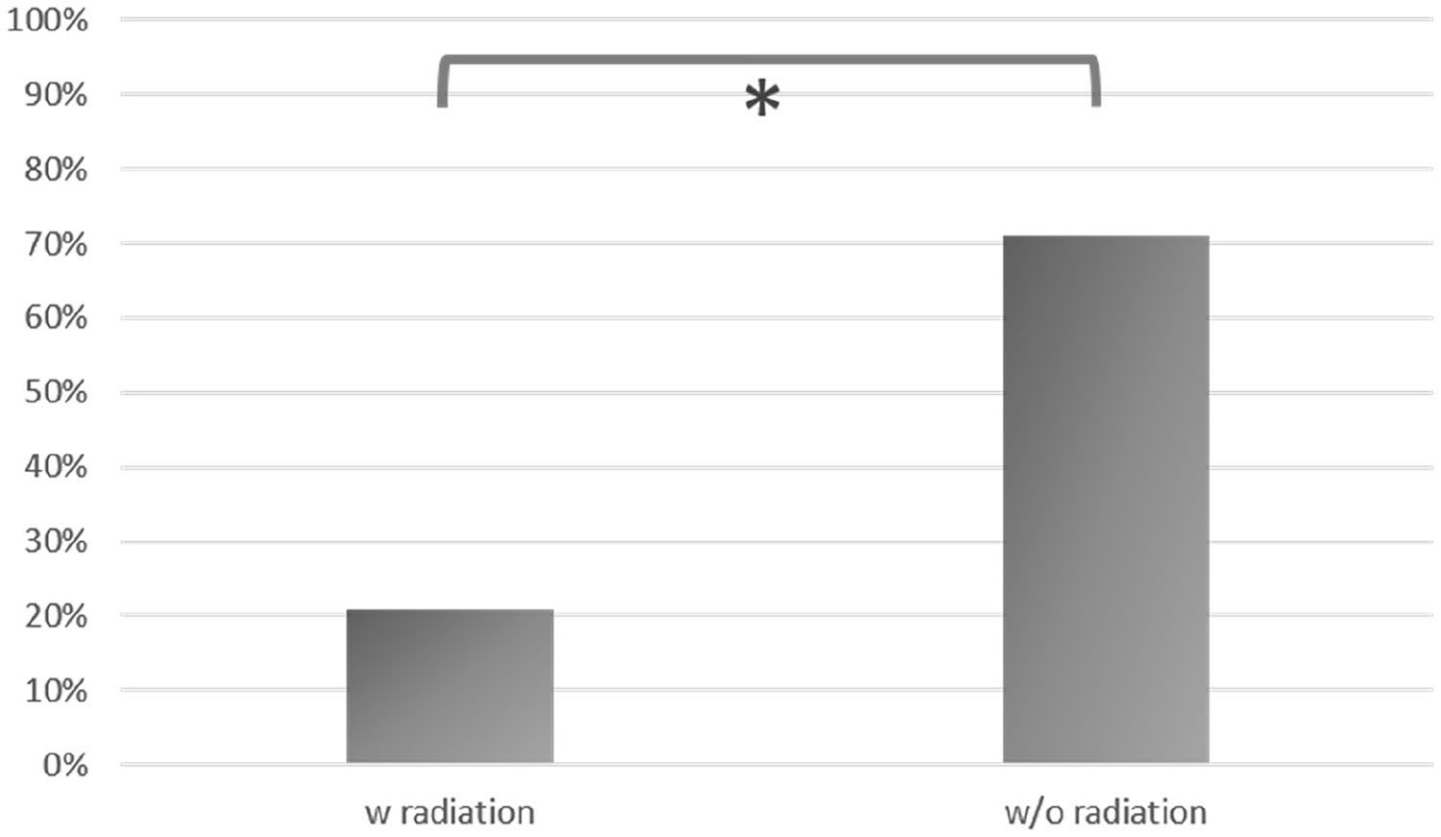
Comparison of the percentage of complete reconstructed breasts using lipofilling for patients with and without radiation

During the treatments, there was one patient with a change of procedure to implant-based reconstruction; in three cases a change to flap reconstruction (1 × DIEP, 2 × Latissimus dorsi flap) was performed. In nine patients, the treatment was stopped unsuccessfully (Figs. 4, 5). They had no further reconstruction at our department (Fig. 6).

Ten women had a history of chemotherapy; one was active smoker. None of the patients had diabetes.

Oil cysts as minor complication were seen in one patient.

Contrasting the two groups, the rate for complete breast reconstruction was significantly higher in Group A (without pre-operative radiotherapy) than in Group B (with pre-operative radiotherapy) (*p* value: 0.016).

The external expander (BRAVA) was used in 11 out of 14 irradiated patients and in nine out of 12 women (11 out of 14 cases) without radiotherapy [17]. The system was worn 6 weeks preoperatively for 8–10 h per day and for 2 weeks postoperatively. No significant difference in achieved complete reconstructions was observed for patients with or without using the Brava system in Group A (*p* value: 1) and Group B (*p* value: 1).

## Discussion

In this study, the influence of radiotherapy for the successful reconstruction of the female breast only by autologous fat transfer is evaluated.

This technique competes with established breast reconstruction procedures, such as perforator flaps or implant-based techniques. Because fat injection-based reconstruction almost always requires multiple procedures (up to nine different surgeries in our own experience) and the outcome is less predictable than with other techniques, only a selected group of women are candidates for this type of reconstruction.

The safety of autologous fat transfer to the breast was investigated in several studies [4–6]. In a previous study, we could demonstrate in a group of 93 women after breast cancer that in a long-term retrospective evaluation with a mean follow-up of 6.7 years, only 1.1% of patients experienced local recurrence after autologous fat transfer to the breast [18].

As the literature for this technique is sparse, comparisons between complete fat injection-based reconstructions and established procedures can hardly be found.

In one of the largest series of lipofilling at the breast, Khouri et al. [17] demonstrated a higher complication rate and more necessary procedures in case of previous radiation.

Heine et al. [19] were able to demonstrate, that skin sensation after fat injection-based breast reconstruction showed more natural values than after perforator flap reconstruction or expander-based techniques.

One of the most crucial factors for reliable in-growth of adipose tissue after lipofilling is the quality, perfusion and elasticity of the recipient tissue. Beneath reduced vascularization and higher proportion of fibrous connective tissue, applied pressure during lipofilling is another relevant factor for successful outcome. Klein et al. examined the pressure increase in the breast tissue during lipofilling procedures. They could demonstrate in a cohort of 36 women with fat transfer to the breast that tissue pressure following lipofilling was rising disproportionately in radiated tissue [20], which can be regarded as a potential risk for viability of the transplanted fat cells [21]. Additional preconditioning of the recipient site with several different treatment options to increase fat graft survival has been investigated in several studies [22]. Orange et al. [23] reviewed thirteen animal studies with five different techniques for preconditioning of the recipient site and found some positive effect on cellular activity (cell proliferation and angiogenesis) by all studies.

Some authors discuss the beneficial effect of the simultaneous use of platelet-rich plasma (PRP) to improve fat engraftment in general [24, 25]. Furthermore, the use of adipose-derived stem cells (ASCs) contributes to adipogenesis and neo-angiogenesis and may represent promising new approaches [26]. In this study, the only preconditioning used for some of the women was an external expander (BRAVA) to enlarge the recipient tissue for fat injection.

Irradiation of the thoracic wall impacts the recipient’s tissue significantly [15, 27]. The influence of previous radiotherapy on the in-growth of transplanted fat has already been investigated in several publications. While some studies about complete breast reconstruction by lipofilling found a negative influence of previous radiation of the recipient tissue compared to non-radiated tissue [17, 20], other authors could demonstrate an advantage of lipofilling as a preliminary treatment before implant-based reconstruction. Debald et al. [13] investigated 40 women with lipofilling procedures after breast conserving procedure or reconstruction, 26 of whom underwent radiotherapy. They found improvement of skin and soft tissue after lipofilling following radiation.

While lipofilling is able to improve soft tissue as an additional tool following breast reconstruction by traditional techniques, complete reconstruction of the breast mound exclusively by lipofilling is essentially dependent on the tissue quality of the recipient area.

In our study, where 95 lipografting procedures on 26 patients (28 breasts) were investigated, we were able to demonstrate the influence of irradiation on lipofilling after mastectomy. We could show that radiotherapy is one of the decisive factors when complete reconstruction by fat grafting is planned following mastectomy.

Furthermore, treatment with an external expander (BRAVA), which was used in 75% (group A) or 79% (group B), had no significant impact on the overall fat survival rate in patients who had undergone radiation prior to lipofilling.

Limitations of this study are the limited number of patients and the retrospective evaluation.

## Conclusion

Successful complete breast reconstruction by lipofilling after previous radiotherapy should be considered as a highly unpredictable procedure, whereby each patient has to be scrutinized individually. Women with an indication for breast reconstruction, especially pre-radiated patients, should always be informed about potential failure or non-successful in-growth of autologous fat transplantation, and, furthermore, should be informed about alternative treatment options for breast reconstruction.
